# A rationally designed optochemogenetic switch for activating canonical Wnt signaling

**DOI:** 10.1016/j.isci.2023.106233

**Published:** 2023-02-19

**Authors:** Seunghwan Lee, Mingguang Cui, Donghun Lee, Kihoon Han, Woong Sun, Dongmin Lee

**Affiliations:** 1 Department of Anatomy, Korea University College of Medicine, Seoul, Republic of Korea; 2 BK21 Graduate Program, Department of Biomedical Sciences, Korea University College of Medicine, Seoul, Republic of Korea; 3 Department of Physics, Korea University, Seoul, Republic of Korea; 4 Department of Neuroscience, Korea University College of Medicine, Seoul, Republic of Korea

**Keywords:** Biochemistry methods, Functional aspects of cell biology, Methodology in biological sciences

## Abstract

Accurate spatiotemporal control of multicellular self-organization by various signaling pathways is essential for developmental stages. In particular, evolutionarily conserved Wnt signaling serves as a major morphogenetic switch to determine the anteroposterior axis of the embryo. Here, we developed a genetically encoded optochemogenetic Wnt switch, named optochemoWnt, by coupling a blue light-inducible CRY2olig and rapamycin-inducible LRP6c clustering. The rationally designed optochemoWnt successfully modulated Wnt signaling with AND-gated patterns and demonstrated an improved signal-to-noise ratio (SNR). The dual-triggered switch provides a safeguard to prevent signal leakage resulting from ambient light sources under general laboratory conditions. OptochemoWnt expands the molecular toolbox available for the fields of developmental biology and tissue engineering. In addition, the AND-gated strategy of optochemoWnt may be used for other biomedical applications that integrate user defined switch elements with Boolean logic gates.

## Highlights

• We developed a genetically encoded Wnt activator termed optochemoWnt
• OptochemoWnt is optochemogenetically triggered by AND logic gating
• The dual-triggered switch reduces signal leakage and enables precise spatial control

## Graphical abstract

**Figure:**
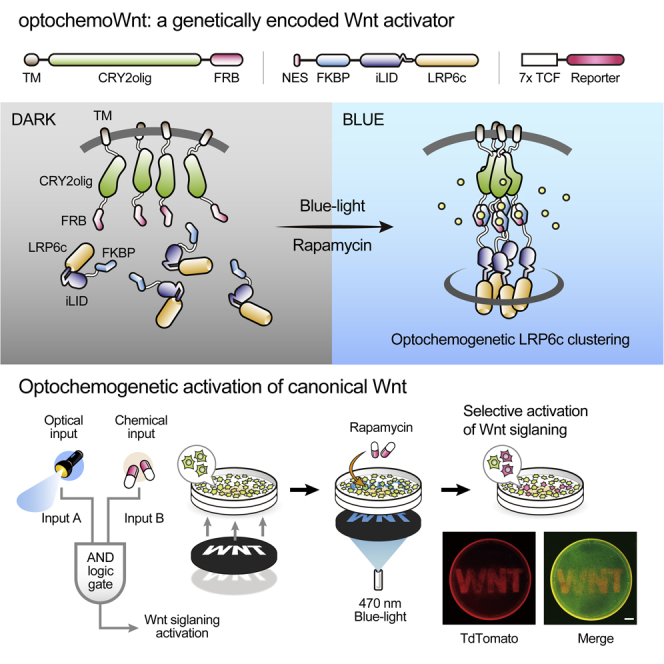

## Introduction

Multicellular self-organization occurs through evolutionarily conserved signaling pathways under precise developmental programming.^1^ In particular, Wnt signaling^2^^,^^3^ is known as a major morphogenetic switch that determines the anteroposterior axis and contributes to asymmetric cell differentiation of rostral and caudal patterning.^4^^,^^5^ Moreover, Wnt signaling plays an important role in adult tissue homeostasis for self-renewal^6^ and is strongly implicated in tumorigenesis,^7^ including colorectal cancer.^8^

Canonical Wnt signaling is tightly regulated by the bimodal states of β-catenin.^9^ The off-state of β-catenin occurs through its constitutive cytoplasmic degradation of related transcription factors.^10^ The ubiquitin-dependent degradation of β-catenin occurs through a destruction complex that includes the tumor suppressors axin, adenomatous polyposis coli, and glycogen synthase kinase 3 (GSK3).^9^ In contrast, the on-state of Wnt signaling is initiated by the extracellular co-binding of secreted glycoprotein Wnt and its specific receptor Frizzled (FZD) and co-receptor LRP.^11^ The trimeric complex of Wnt/FZD/LRP inhibits the degradation of β-catenin by inhibiting the activity of the destruction complex. Stabilized β-catenin translocates to the nucleus and promotes target gene expression with interactions of natural transcription factors such as lymphoid-enhancing factor-1 (LEF-1)^12^ and T cell factor (TCF).^13^

The multi-step pathway of canonical Wnt signaling just described allows broad strategy to design genetically encoded Wnt activators under user-defined control. Specially, clustering of LRP6c presents simple and effective induction of Wnt signaling regardless of the ligand-receptor binding.^14^^,^^15^ Clustering of LRP6c could be implemented by various protein-protein binding methods such as chemogenetics or optogenetics. Optogenetic complementation using split proteins has emerged as a biological breakthrough, because of remote control and precise spatiotemporal resolution. Light-responsive proteins found in plants or microorganisms can be used as a photo switch. Above all, Light-Oxygen-Voltage (LOV)^16^ and cryptochrome (CRY2)^17^ are two of the most popular non-opsin photoswitches and have a common feature of responding to blue-light.^18^ To our knowledge, two optogenetic Wnt activating systems have been developed by introducing blue light-inducible CRY2 oligomerization^19^ and heterodimerization,^20^ respectively. Although these optogenetic systems show reliable induction of Wnt signaling, they still have some innate limitations including signal leakage and shielding problems.

In this study, we generated a dual-triggered optochemoWnt under AND gate control to circumvent the innate problems of optogenetics. OptochemoWnt is a rationally designed Wnt signaling activator, as its name suggests, and offers the synergistic benefits of optogenetic and chemogenetic systems. The dual-mode control of optical systems not only provides accurate spatiotemporal manipulation, but also offers a solution to the problem of leakage caused by the ubiquitous nature of light. OptochemoWnt is a versatile tool for the fields of developmental biology and tissue engineering by enabling user-defined reprogramming of the developmental process.

## Results

### Rational design of the optochemogenetic Wnt activating system

**Figure 1:**
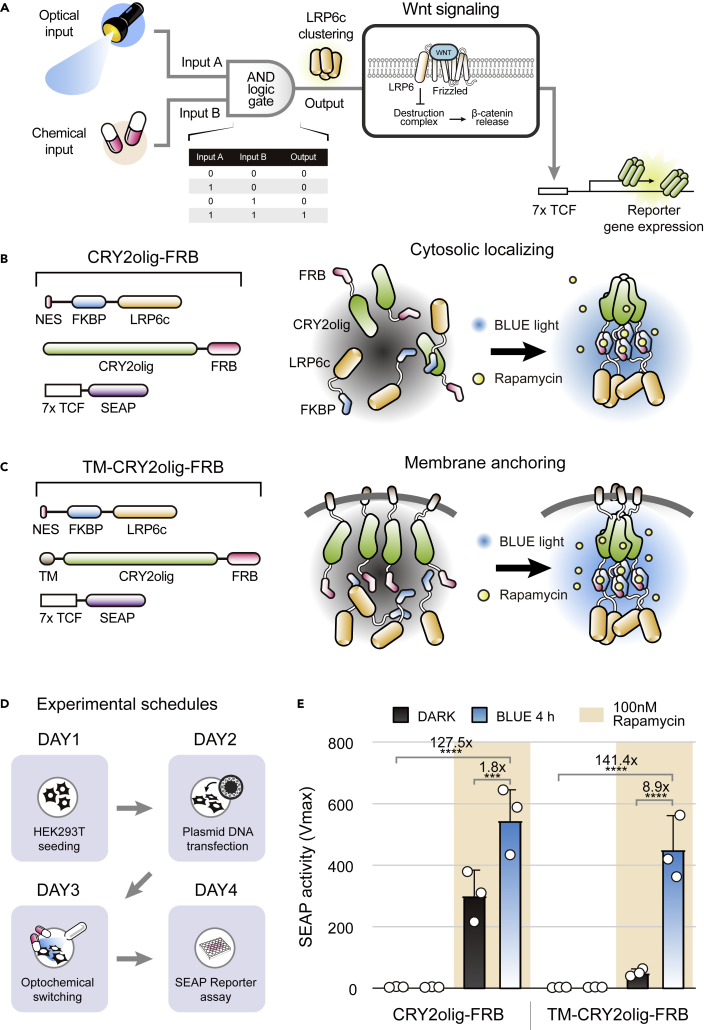
Rational design of a dual-triggered Wnt signaling activator (A) Schematic diagram depicting a simple workflow of the Wnt signaling activator known as optochemoWnt. Canonical Wnt signaling can be activated by the clustering of LRP6c, c-terminal cytosolic part of the Wnt co-receptor. The clustering of LRP6c as a final output is modulated by dual-triggers based on an AND logic gate. (B and C) Construct configurations and schematic workflow of (B) cytosolic localizing and (C) membrane anchoring optochemoWnt. The only difference between the two prototypes was the absence or presence of a transmembrane module within the N-terminus of the first component. The first component contains the optical actuator of CRY2 and one of the chemical linkers, FRB. The second includes another chemical linker, FKBP, and LRP6c. The light-induced oligomerization of CRY2olig is transmitted into the clustering of LRP6c in the presence of rapamycin-dependent heterodimerization. (D) Four-days scheduled experimental time-line for optochemoWnt. Seeding of HEK293T cells (Day 1), transfection of plasmid vectors (Day 2), optical and chemical stimulation (Day 3), and SEAP-based reporter assay (Day 4) were done at 24-h intervals. (E) Blue light- and rapamycin-dependent Wnt activation of cytosolic localizing and membrane anchoring optochemoWnt. Wnt activity of optochemoWnt was monitored using a SEAP-based assay. Membrane anchoring optochemoWnt shows a higher efficiency of Wnt activation and a clearer AND-gated pattern compared with the cytosolic localizing construct. For activation of optochemoWnt, a 4 h illumination (duty cycle: 2 s on/58 s off) with blue light (470 nm) and treatment with 100 nM rapamycin was carried out. Data were collected from three biologically independent experiments and are presented as means ± S.D. The white circles represent the measured value of individual experiments. Statistical analysis was done using a two-way ANOVA followed by Tukey’s multiple comparisons test (∗∗∗p < 0.001; ∗∗∗∗p < 0.0001).

Inspired by single-triggered optoWnt,^19^^,^^21^ we developed a dual-triggered Wnt activating system based on an AND logic gate (Figure 1A). Because of its optochemogenetic feature, we designated this two-component system optochemoWnt. The first component, which serves as a light-induced homo-oligomerizer for rapid protein clustering, was prepared by introducing blue light-induced CRY2olig (E490G)^22^ with rapid clustering kinetics as an engineered variant of CRY2. Similar to optoWnt, the second component of optochemoWnt includes LRP6c, a key regulator of Wnt signaling. Separated two components were chemically coupled by heterodimerization of rapamycin-dependent FKBP/FRB. In the presence of rapamycin, the blue light-dependent oligomerization of CRY2olig may be transmitted to another component to generate LRP6c clustering. Based on our initial design, we cloned two cytosolic components of optochemoWnt (Figure 1B) and tested the prototype optochemoWnt using a secreted embryonic alkaline phosphatase (SEAP) luminescence assay (Figure 1D). As a result, we observed significant rapamycin-dependent Wnt activation of optochemoWnt, but moderate levels of light-independent noise (Figure 1E, left panel). We hypothesized that the highly free arrangement of cytosolic optochemoWnt would produce nonspecific, inter-component aggregation, resulting in the nonspecific clustering of LRP6c. To minimize irregularly oriented arrangements, we anchored the CRY2olig component to the plasma membrane using a transmembrane domain of the platelet-derived growth factor receptor (Figure 1C). Consistent with our hypothesis, the membrane immobilization of CRY2olig significantly reduced nonspecific Wnt activity in the DARK control, while increasing Wnt activity in the group treated simultaneously with blue light and rapamycin (Figure 1E, right panel).

### Engineering and optimization of optochemoWnt

**Figure 2:**
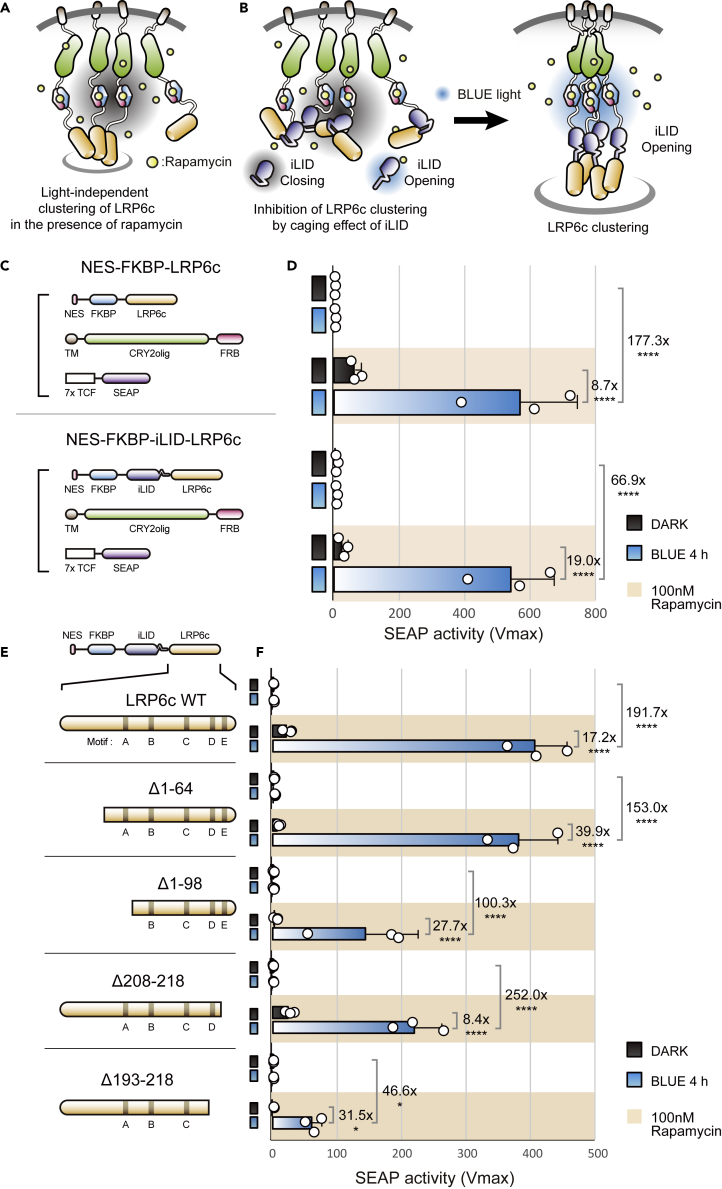
Engineering and optimization of optochemoWnt (A) Graphical illustration showing the light-independent, nonspecific clustering of LRP6c resulting from overcrowded optochemoWnt onto with planar arrangements. (B) Caged optochemoWnt by integrating the light-induced photo switch, iLID. Folded and closed conformation of DARK-stated iLID minimizes external accessibility of LRP6c (left), whereas blue light-induced opening of iLID maximizes external accessibility of LRP6c (right). (C) Plasmid configuration of opened (upper; NES-FKBP-LRP6c) and caged optochemoWnt (lower; NES-FKBP-iLID-LRP6c). (D) Optochemogenetically induced SEAP expression of opened (upper) and caged (lower) optochemoWnt in HEK293T cells. Summary graph showing increased fold-change of caged optochemoWnt activity (19.0x) compared with the previous one (8.7x). The white circles represent individual measurements from three independent experiments. The data are presented as means ± S.D. Two-way ANOVA followed by Tukey’s multiple comparisons test. (∗∗∗∗p<0.0001). (E) OptochemoWnt variants with n- or c-termini truncated LRP6c (Δ1-64, Δ1-98, Δ208-218, and Δ193,218) based on the spatial distribution of LRP6c motifs (motif A, B, C, D, and E). (F) Optochemogenetic SEAP expression of five optochemoWnt truncated variants including WT. Summary graph shows optochemogenetically induced folds of five optochemoWnt variants. Loss of any motif of optochemoWnt generates a significant reduction in Wnt activity, whereas N-terminal trimming of LRP6c (Δ1-64) without deleting the motif exhibits a higher fold (39.9x) change compared with the WT (17.2x). The measured values were collected from three biologically independent samples. The white circles represent individual measurements. The data are presented as means ± S.D. from three independent experiments. Two-way ANOVA with Tukey’s multiple comparisons test was used to assess the significance of the differences between the blue light/rapamycin group and the other groups. ∗p < 0.05, ∗∗∗∗p < 0.0001.

To further reduce the light-independent, nonspecific background of optochemoWnt (Figure 2A), we introduced a novel blue light-responsive photo switch known as iLID.^23^ In our previous studies, iLID was successfully used to control the proteolytic activity of a coupled protease substrate through steric masking of its Jα helix domain opening.^24^^,^^25^ We predicted that the off-state of iLID-LRP6c could mask the LRP6c domain like a closed hairpin (Figure 2B, left panel), thereby hindering LRP6c activity for the Wnt signaling pathway, whereas the on-state of iLID-LRP6c would provide free accessibility to LRP6c through light-induced exposure of the Jα helix (Figure 2B, right panel). In a test of optochemoWnt with iLID (Figure 2C), we observed higher Wnt activity in the iLID group (Figure 2D), indicating that light-inducible steric occlusion of iLID contributes to reducing the noise level of optochemoWnt.

Because the cytosolic domain of LRP6 is a key actuator of Wnt signaling, engineering LRP6c and its neighboring modules is the most direct way to improve the performance of optochemoWnt. LRP6c contains five evolutionally conserved motifs (PPPSP), which serve as a phospho-regulated docking sites for the disruption of the complex protein axin.^26^^,^^27^ We hypothesized that steric occlusion of iLID-LRP6c activity would be further strengthened by trimming the terminal region of LRP6c with the exception of five motifs. Based on this idea, we constructed four truncated mutants of LRP6c (Δ1-64, Δ1-98, Δ208-218, and Δ193-218) by trimming the n- or c-termini (Figure 2E). Of interest, removal of more than a single motif significantly reduced Wnt activity, but removal of only the terminal region without deletion of any motifs (Δ1-64) resulted in an enhanced signal change, which suggests that shortening the extra n-terminal region increases the steric masking power of iLID-LRP6c (Figure 2F).

Before moving toward the intensive characterization of optochemoWnt, we investigated whether the clustering of CRY2olig is essential for the function of optochemoWnt. To verify the importance of CRY2olig within the optochemoWnt system, we introduced a D387A CRY2olig inactive mutant^28^ (Figure S1A), which lacks a binding affinity of the flavin adenine dinucleotide (FAD), a cofactor of CRY2olig. The inactive mutant did not exhibit Wnt activity regardless of light illumination (Figure S1B), demonstrating the indispensability of CRY2olig as the primary clustering actuator.

### Characterization of optochemoWnt

To refine the stimulation protocol of optochemoWnt, we established gene expression profiles for various durations and concentrations of blue light and rapamycin, respectively (Figure S2A). First, we measured SEAP levels during blue light exposure for up to 16hat 100 nM rapamycin. As expected, gene expression correlated positively with the duration of blue light exposure (Figure S2B) and 16 h of blue light exposure induced a 77.8-fold increase compared with the DARK control (Figure S2B). Next, we examined chemically induced gene expression profiles based on the concentration of rapamycin under blue light for 16 h. Similar to the optical induction test, the expression of SEAP increased concomitantly with increasing rapamycin concentration (Figure S2C). The maximum expression levels peaked at rapamycin concentrations above 100 nM (Figure S2C). Based on these results, we established a stimulation protocol of optochemoWnt using 16 h of blue light exposure and 100 nM rapamycin.

**Figure 3:**
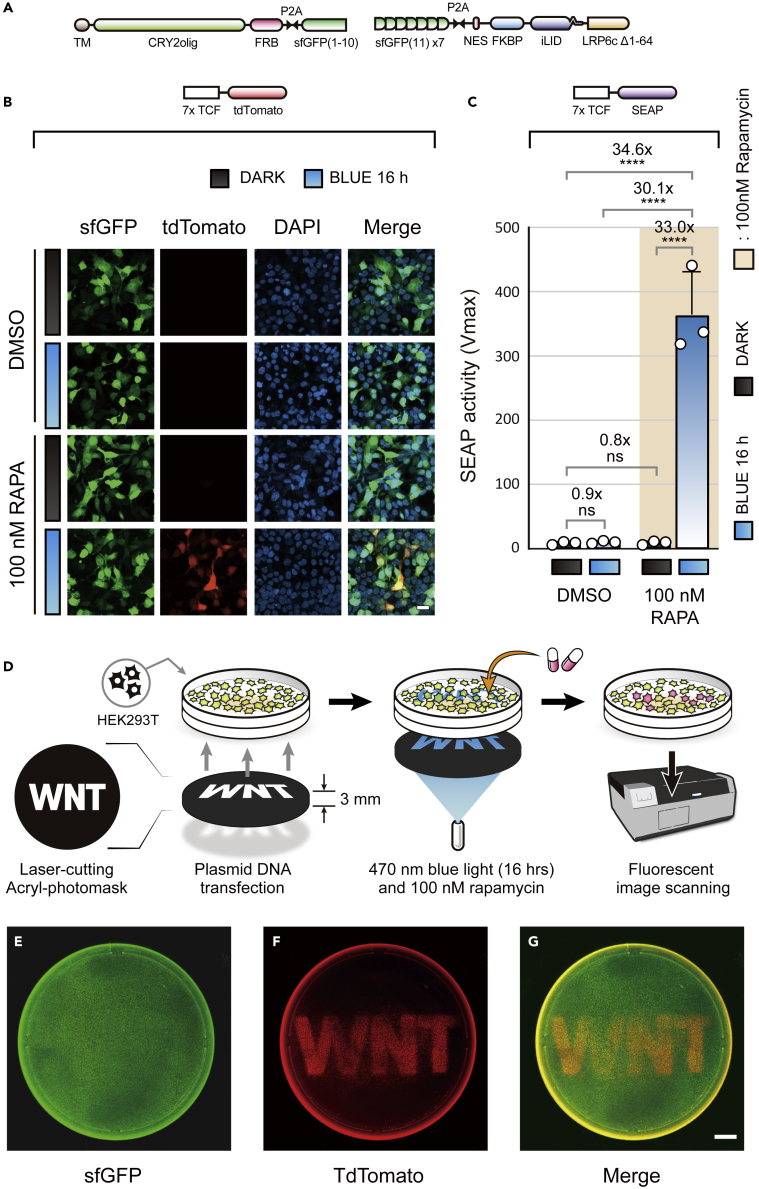
Characterization of AND-gated optochemoWnt (A) Configuration of the transfected plasmids of optochemoWnt. (B) Representative confocal images of optochemoWnt. A splitGFP signal was used as a co-transfection marker and tdTomato as a reporter of Wnt activity. DAPI staining of the nucleus indicates the entire field distribution of the cells. Scale bar, 20 *μ*m. (C) SEAP quantification of optochemoWnt. Summary graphs exhibit AND-gate patterns with increasing SEAP activity upon blue light and rapamycin exposure. Mean values were calculated from three biological replicates and presented as means ± S.D. The white circles represent individual measurements from three independent experiments. Two-way ANOVA followed by Tukey’s multiple comparisons test. (∗∗∗∗p < 0.0001). (D) Sequential cartoons showing the experimental procedures of blue light-induced spatial expression of optochemoWnt through a photomask. (E–G) Fluorescent images of tdTomato (middle) as a reporter and constitutive splitGFP (left) as a co-transfection marker. The right panel is a merged image of splitGFP and tdTomato. Scale bar, 10 mm.

Using the above stimulation protocol, we validated the performance of optochemoWnt using fluorescence- and SEAP-based assays. For the fluorescence assay, we introduced a split super-folder GFP (sfGFP) system^29^ to selectively label cells that co-express the two components of optochemoWnt (Figure 3A). Each fragment of the split sfGFP system, which consisted of a single 1–10 β-strand and seven tandem 11th β-strands, was integrated into two plasmid vectors through a self-cleaving P2A peptide derived from porcine teschovirus-1.^30^ Although bicistronic insertion of split sfGFP reduces the number of plasmid vector for transfection, it also inevitably increases the size of individual plasmid. In mammalian cells, there are some studies that the increased size of DNA may affect the expression or activity of recombinant proteins.^31^ To rule out this undesirable effect, we evaluated whether the performance of the optochemoWnt can be affected by the bicistronic introduction of sfGFP (Figure S3A). As a result, expressed SEAP levels were quite similar between the photochemical Wnt system introduced with sfGFP and the system without a fluorescent marker system (Figure S3B), indicating that the bicistronic system for co-expression of split sfGFP had little effect on the activity of optochemoWnt.

To measure the performance of optochemoWnt using four conditions of DARK/BLUE or DMSO/rapamycin, we transfected optochemoWnt and 7 × TCF-tdTomato into HEK293 T cells. In the DARK control, there was no detectable tdTomato expression regardless of rapamycin treatment; however, following blue light illumination, tdTomato was observed in the rapamycin-treated group (Figure 3B). The typical AND-gated pattern of tdTomato expression indicated reliable Wnt induction of optochemoWnt under the control of dual-triggered stimulation. In addition, we verified the performance of optochemoWnt using SEAP as a reporter. Similar to the results of the fluorescence assay, SEAP was only observed under conditions in which blue light and rapamycin were administered simultaneously (Figure 3C).

Precise gene expression with high spatial resolution is an outstanding feature of optogenetics compared with other induction systems. Therefore, we visualized the spatial resolution of optochemoWnt through a lettered photomask which was perforated with “WNT.” We transfected constructs of optochemoWnt and 7xTCF-tdTomato into HEK293 T cells. Blue light was illuminated through the photomask precisely on the cellular regions and induced photomask-dependent expression of tdTomato (Figure 3D). As the result, the scanned fluorescent image clearly displayed randomly distributed green fields (Figure 3E) and the red “WNT” initials (Figure 3F), which indicated the precise optogenetic induction of optochemoWnt at high spatial resolution.

### Benchmarking of genetically encoded Wnt activators

**Figure 4:**
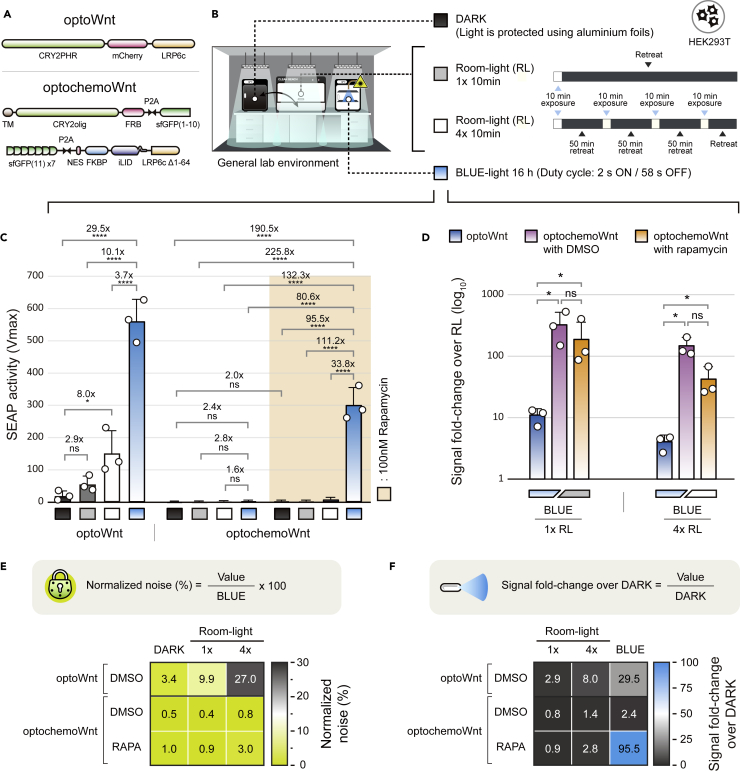
Benchmarking of optoWnt and optochemoWnt (A) Plasmid configurations of single input-triggered optoWnt and dual input-triggered optochemoWnt for benchmarking tests in terms of signal leakage and fold-change. (B) Schematic depicting four types (DARK, Room light single trial, Room light four trials, and blue light) of conditions of light stimulation. For the DARK condition, the cells were wrapped with tight aluminum foil. In each trial under room-light conditions, the cells were left on an external clean bench with all of the lights illuminated for 10 m, then the cells were placed into DARK conditions for 50 min. The room-light condition mimics ordinary light exposure during general culture procedures, such as microscopic monitoring, media changes, and reagent administration. (C) The benchmark test of optoWnt and optochemoWnt under the four types of light activation conditions described above. The values are expressed as means ± S.D. and white circles are presented as individual measurements. Statistics included a one-way ANOVA(optoWnt) and two-way ANOVA(optochemoWnt) followed by Tukey’s multiple comparisons test. (ns = not significant, ∗p < 0.05, ∗∗∗∗p < 0.0001). (D) Signal fold-change over RL (The values of Y axis is log scale). The signal fold-change exhibits the execution efficiency of each construct (blue-colored bar: optoWnt, purple-colored bar: optochemoWnt with DMSO, and orange-colored bar: optochemoWnt with rapamycin). The values are expressed as means ± S.D. and white circles are presented as individual measurements. Statistics included a one-way ANOVA(optoWnt) and two-way ANOVA(optochemoWnt) followed by Tukey’s multiple comparisons test. (ns = not significant, ∗p < 0.05). (E) An equation (upper gray box) for the normalization of noise ratio. The normalized noise ratio was introduced to quantify undesirable leakage from the light conditions, except for BLUE light, and was calculated by dividing the DARK or Room-light value by the BLUE light value. A color-coded heatmap (bottom panel) represents the normalized noise ratio of optoWnt and optochemoWnt. (F) Equation (upper gray box) for a signal fold-change over DARK. The signal fold-change represents the performance efficiency of each construct. A color-coded heatmap (bottom panel) indicates the extent of signal fold changes in optoWnt and optochemoWnt. The results (C, D, E, and F) are representative of three independent experiments.

The most innovative part of optochemoWnt is the dual-triggered activation system, in which Wnt signaling cannot be activated even under prolonged exposure to light unless the controllable system is turned on following treatment with rapamycin. To demonstrate the stability of optochemoWnt under room-light conditions, we directly benchmarked optochemoWnt and optoWnt^19^^,^^21^^,^^32^ (Figure 4A), the first generation Wnt activator system, which is triggered only by blue light exposure. The room-light (RL) condition was established using a 10-min exposure on a clean bench with the lighting turned on, similar to typical cell culture protocols, such as media replacement or microscopic monitoring (Figure 4B). Based on similar lighting conditions in the general lab environment, we benchmarked both Wnt activators (Figure 4C). Single-triggered optoWnt was readily induced by the RL conditions, whereas dual-triggered optochemoWnt was resistant, despite repetitive trials of RL (Figure 4C). In the case of optochemoWnt, rapamycin-dependent signal increase in DARK (Figure 4C; DARK_Rapamycin_/DARK_DMSO_ = 2.0x) and blue light-dependent signal increase in DMSO (Figure 4C; BLUE_DMSO_/DARK_DMSO_ = 2.4x) were almost negligible, demonstrating the perfect AND-gated pattern of optochemoWnt. In addition, we directly compared the signal fold-change of optoWnt and optochemoWnt depending on both conditions of RL (BLUE/RL 1x or BLUE/RL 4x). As expected, optochemoWnt showed significantly higher fold-changes than optoWnt under both BLUE/RL conditions (Figure 4D).

To further determine the performance of each system, we also introduced normalized noise and again measured the fold-change over DARK (Figures 4E and 4F). Normalized noise (Figure 4E) is the relative percentage of the noise value divided by the value of BLUE conditions. The signal fold-change over DARK (Figure 4F) is the relative increase of the signal value divided by the value of the DARK control. That is, a lower normalized noise and a higher fold-change in signal indicate better performance of the Wnt activator. In a test of the single or four trials under room-light conditions, the single input-triggered optoWnt exhibited a significantly higher normalized noise (Figure 4E; 9.9 or 27.0%, respectively) and fold-change in signal (Figure 4F; 2.9x or 8.0x, respectively) of Wnt activity compared with DARK conditions, suggesting that optoWnt is very vulnerable to ambient lighting. The normalized noise of optochemoWnt was only 0.4%–0.8% in the absence of rapamycin and 0.9%–3.0% in its presence (Figure 4E), whereas the fold-change of optochemoWnt was 0.8- to 1.4-fold in the absence of rapamycin and 0.9- to 2.8-fold in its presence (Figure 4F). These data indicate that the optochemoWnt system is precisely controlled by dual-triggered inputs and the dual input-triggered optochemoWnt is more suited for *in vitro* culture applications than the single input-triggered optoWnt.

## Discussion

In this study, we engineered a genetically encoded molecular switch, named optochemoWnt, to activate canonical Wnt signaling with dual-triggers based on an AND logic gate. To date, several optogenetic techniques, such as optoWnt^19^^,^^21^ and optoLRP6opt,^20^ have been developed to regulate Wnt activation with a single trigger. In the case of optoWnt, homo-oligomeric clustering of CRY2 Photolyase Homology Region (CRY2PHR) induces clustering of LRP6c, whereas optoLRP6opt utilizes membranous accumulation of LRP6c via an optical interaction of plasma membrane-targeted CRY2PHR and cryptochrome interacting basic-helix-loop-helix. Although these single-input optogenetic systems show precise control of Wnt signaling, they have innate limitations, such as a constitutive leakage and limited accessibility resulting from the ubiquitous nature of light. To overcome the limitations of the single-input system, we designed a dual-triggered optochemogenetic system with two-component architecture. For the first component, CRY2olig, an engineered variant of CRY2PHR,^22^ and FRB, one of a pair of rapamycin-dependent heterodimers, were linked together. The second component contained FKBP, another rapamycin-dependent heterodimer pair and LRP6c. The separate two-component system effectively prevented constitutive leakages in the absence of inter-componental coupling. User-controlled rapamycin treatment and blue light exposure of optochemoWnt enabled optogenetic clustering of CRY2olig, which is chemically transmitted to LRP6c located in other components. The rapamycin-dependent interaction of FKBP/FRB acts as a molecular gearbox that turns the system on and off.

Structurally, optochemoWnt has several characteristic differences compared with optoWnt and optoLRP6opt. First, optochemoWnt includes an additional optogenetics module (iLID). In previous optogenetic applications, iLID was predominantly used to form a blue light-induced heterodimer with its binding partner, stringent starvation protein B (SspB). However, in this study, the light-induced intramolecular transformation of iLID acts as a structural organizer to induce a spatial disorientation without SspB. LRP6c contains five conserved motifs that regulate Wnt signaling. We demonstrated that truncated LRP6c exhibits enhanced SNR compared with the wildtype. Although it is not certain, LRP6c and neighboring iLID sequences will be important for generating a caging effect of optochemoWnt. We could consider another caging strategy, such as Latching Orthogonal Cage Key pRotein (LOCKR),^33^ that covers the motif region.

The Wnt signaling pathway is a key regulator in neural tube formation at the embryonic developmental stage.^34^ Considering the importance of Wnt signaling as a morphogenetic regulator during early development, the future direction and application of optochemoWnt are very straightforward. For example, optochemoWnt can be applied to many applications in which Wnt signaling needs to be precisely modulated. In particular, the advantage of optochemoWnt is evident in long-term culture *in vitro*. Daily culture procedures, such as cell medium changes and microscopic monitoring, can cause repeated light exposure to the optogenetic WNT activation system and single-input optogenetic systems are very vulnerable to these effects. However, dual-triggered optochemoWnt will provide a reliable safeguard to prevent noise accumulation during long-term culture, such as neuronal or stem cell culture. In addition, optochemoWnt may be used for self-organized tissue engineering. Wnt signaling contributes to morphogenesis in the development of various tissues. *In vitro* tissue engineering requires the fine-tuning of cellular signaling including Wnt. For example, the determination of orientation in early neural tubes results from spatial gradients of morphogens, which are induced by a conserved Wnt signaling gradient.^35^ Several tools, such as microfluidic-controlled stem cell regionalization,^36^ have been developed to generate chemical gradients for Wnt signaling activation; however, gradient formation of Wnt signals using microfluidic systems has the disadvantage of requiring complicated microfluidic control devices and the impossibility of fine-tuning at the cellular level. Optogenetic systems with accurate spatial resolution have the advantage of being able to implement signal gradients in a much more sophisticated manner compared with drug-based systems.

In conclusion, we successfully developed optochemoWnt based on an AND logic gate and demonstrated that it exhibits enhanced SNR and reliable control of Wnt signaling. Co-stimulation with blue light and rapamycin results in reduced signal leakage and easy accessibility during *in vitro* culture. The dual-triggered strategy of optochemoWnt to modulate Wnt activity will lead to the fabrication of synthetic molecular circuits and expand the scope of optogenetics in the field of developmental biology and tissue engineering.

### Limitations of the study

Increased system sizes over single-triggered Wnt activators are unavoidable trade-offs for implementing dual-triggered systems under controls of optical and chemical stimulation. The increased size of optochemoWnt transcripts may result in low efficiency of transduction or transfection when transiently expressing optochemoWnt. In particular, AAV viruses with a small loading capacity may cause difficulties for generating viral constructs of optochemoWnt. To reduce the number of transfection vectors, we could design a bicistronic transcript by introducing 2a self-cleavable peptides, such as P2A.^30^ In addition, a minimalization strategy focused on the longest CRY2olig or alternative clustering inducers, such as HOTag,^37^ may also be considered.

## STAR★Methods

### Key resources table

**Table:** 

REAGENT or RESOURCE	SOURCE	IDENTIFIER
**Bacterial and virus strains**		

DH5α Chemically Competent *E. coli*	Enzynomics	Cat#CP010

**Chemicals, peptides, and recombinant proteins**		

Dimethyl sulfoxide (DMSO)	sigma	Cat#D8418
Fetal Bovine Serum (FBS)	Gibco	Cat#16000-044
Rapamycin	TOCRIS	Cat#1292
Para-Nitrophenyl phosphate (PNPP) substrate tablets	Thermo Scientific	Cat#34047
Pierce^TM^ PNPP substrate kit	Thermo Scientific	Cat#37620
L-Homoarginine	Alfa Aesar	Cat#H27387

**Experimental models: Cell lines**		

Human: HEK293T	ATCC	ATCC#: CRL-11268

**Recombinant DNA**		

Selected plasmid sequences are listed in Table S1	Our lab	Table S1

**Software and algorithms**		

ImageJ	National Institutes of Health, USA	http://imagej.nih.gov/ij/
Zeiss confocal microscope application Zen blue	Zeiss	N/A

### Resource availability

#### Lead contact

Further information and requests for resources and reagents should be directed to and will be fulfilled by Dongmin Lee (overcode@korea.ac.kr) who is the lead contact.

#### Material availability

All plasmids, equipment, and blue light conditions used in this study will be shared by the lead contact upon request.

### Experimental model and subject details

#### Cell lines

HEK293T cells were obtained from the AmericanType Culture Collection and cultured in Dulbecco’s modified Eagle’s medium (DMEM) (WELGENE, Republic of Korea, LM001-05) supplemented with 10% FBS (Gibco, USA [16000-044]) and 1% penicillin-streptomycin (Gibco, USA, 15140-122).

#### Microbe strains

DH5α bacteria were used for cloning and were cultured in Luria-Bertani media with corresponding antibiotics after transformation with recombinant plasmid in a 37°C incubator.

### Method details

#### Design and construction of plasmid vectors

The CRY2PHR-mCh-LRP6c plasmid vector was obtained from Addgene (42960) and the coding sequences of FKBP and FRB were synthesized (Cosmo Genetech, Republic of Korea). We amplified the 7xTCF sequence from the 7TGC vector (Addgene 24304). The PCR products were generated using combinations of restriction enzymes. The CRY2olig sequence was generated by site-directed mutagenesis (E490G) of CRY2PHR. The transmembrane domain (TM) was amplified from the Cal-Light vector (Addgene 92390) and cloned into the CRY2olig-FRB backbone. We acquired coding sequences of spGFP from pcDNA3.1-GFP (1-10) (Addgene 70219) and pHRm-NLS-dCas9-GFP11x7-NLS-P2A-BFP-NLS (Addgene 70224). All reagents for cloning were purchased from New England BioLabs (USA). All cloned plasmid vectors were verified by DNA sequencing (Macrogen, Republic of Korea).

#### HEK293T cell and culture and DNA transfection

HEK293T cells were cultured in Dulbecco’s modified Eagle’s medium (DMEM) (WELGENE, Republic of Korea) containing 10% FBS (Gibco, USA) and 1% penicillin-streptomycin (Gibco, USA). The cells were maintained at 37°C in a 10% CO_2_ atmosphere. The cell culture plates were precoated with 1 mg/ml poly-D-Lysine hydrobromide (PDL) solution for 1 h. Once ∼90% confluent was reached, the medium was removed and the cells were treated with 0.25% Trypsin-EDTA (Gibco, USA) for 2 min. Detached cells were collected for counting and the cells were plated at a density of 1 × 10^5^ cells per well in 24-well plates. Each DNA construct was transfected into cells using a jetOPTIMUS (Polyplus, France) transfection kit as described in the user manual. The total volume of DNA used for transfection was 0.25 *μ*g and the reagent was 0.25 *μ*l per well in a 24-well plate.

#### Light illumination and rapamycin treatment

One day after transfection, the cells were exposed to blue light (450 nm) for 16 h. The blue LED array for illumination was installed inside a 37°C incubator containing 10% CO_2_. The cycle of blue light was controlled by an electronic timer (IRT16-D, Han Seung) with 2 s on and 58 s off. In our experimental setup, the measured intensity of the blue light was 5.8 mW/cm^2^ using a power meter (PM100D, ThorLabs). To minimize the leakage of optochemoWnt, transfected cell plates were tightly wrapped using aluminum foil. Rapamycin was purchased from Tocris Bioscience (UK). Before blue light illumination, 100 nM rapamycin was added.

#### SEAP chemiluminescence assay

For the quantification of reporter gene expression, we utilized a SEAP chemiluminescent assay. The reaction buffer was composed of 5.1 M diethanolamine (Thermo, USA), PNPP substrate tablet (Thermo, USA), L-homoarginine (Alfa Aesar, USA), and 10 mM MgCl_2_ (SG-BIO, Republic of Korea). Before adding the reaction buffer, the samples were preheated at 65°C for 1 h to inhibit endogenous alkaline phosphatase activity. Reaction solution (100 *μ*l) and samples (40 *μ*l) were mixed and carefully added to 96-well plates to prevent bubbles and incubated at 37°C for 10 min. After incubation, PNPP (p-nitrophenyl phosphate) solution was added to determine the kinetics using a microplate reader (Infinite F50, TECAN) at 405 nm absorbance in 30 s intervals for 1 h. The Vmax value was calculated using TECAN Magellan software.

#### Imaging processing

Immobilized cells on coverslips were fixed with 4% paraformaldehyde (CUREBIO) for 30 min and then rinsed three times with 1x PBS. For nuclear staining, the cells were incubated with 300 nM DAPI (Invitrogen, USA, D1306) and rinsed with 1x PBS. The stained cells were mounted on a slide glass using Crystal mount solution (Biomeda USA, M02). Confocal images were obtained using an LSM800 confocal microscope (Zeiss) with a 40x/0.8 M27 objective lens. For the photomask experiment in Figure 3, large-scale cell culture dish images were scanned using a fluorescence image scanner (Typhoon FLA 9500, GE Healthcare).

### Quantification and statistical analysis

The data are presented as mean values ± S.D. of three independent biological repeats within the same experiment. For comparisons between groups, the data were analyzed by a one-way analysis of variance (ANOVA) or a two-way ANOVA followed by Tukey’s multiple comparisons test. The reported p-values from the ANOVA analysis are the adjusted p-values. All statistical analyses were performed using Microsoft excel or GraphPad Prism software. The adjusted p-values are reported using the symbols: ns = not significant, ∗ = p < 0.05, ∗∗ = p < 0.01, ∗∗∗ = p < 0.001, ∗∗∗∗ = p < 0.0001.

## Data Availability

• All data reported in this paper will be shared by the lead contact upon request. • Any additional information required in this paper is available from the lead contact upon request. • This paper does not report original code.

## Supplemental information

Document S1. Figures S1–S3

Table S1. DNA and amino acids sequence infromation of selected plasmid vectors, related to STAR methods
