# Supplementary material for: A rationally designed optochemogenetic switch for activating canonical Wnt signaling
Source: iScience. 2023 Feb 19;26(3):106233. doi: 10.1016/j.isci.2023.106233 (PMC10006827; doi:10.1016/j.isci.2023.106233)
Supplement: Document S1. Figures S1–S3 [file mmc1.pdf]

iScience, Volume *26*

Supplemental information

A rationally designed optochemogenetic switch for activating canonical Wnt signaling

Seunghwan Lee, Mingguang Cui, Donghun Lee, Kihoon Han, Woong Sun, and Dongmin Lee

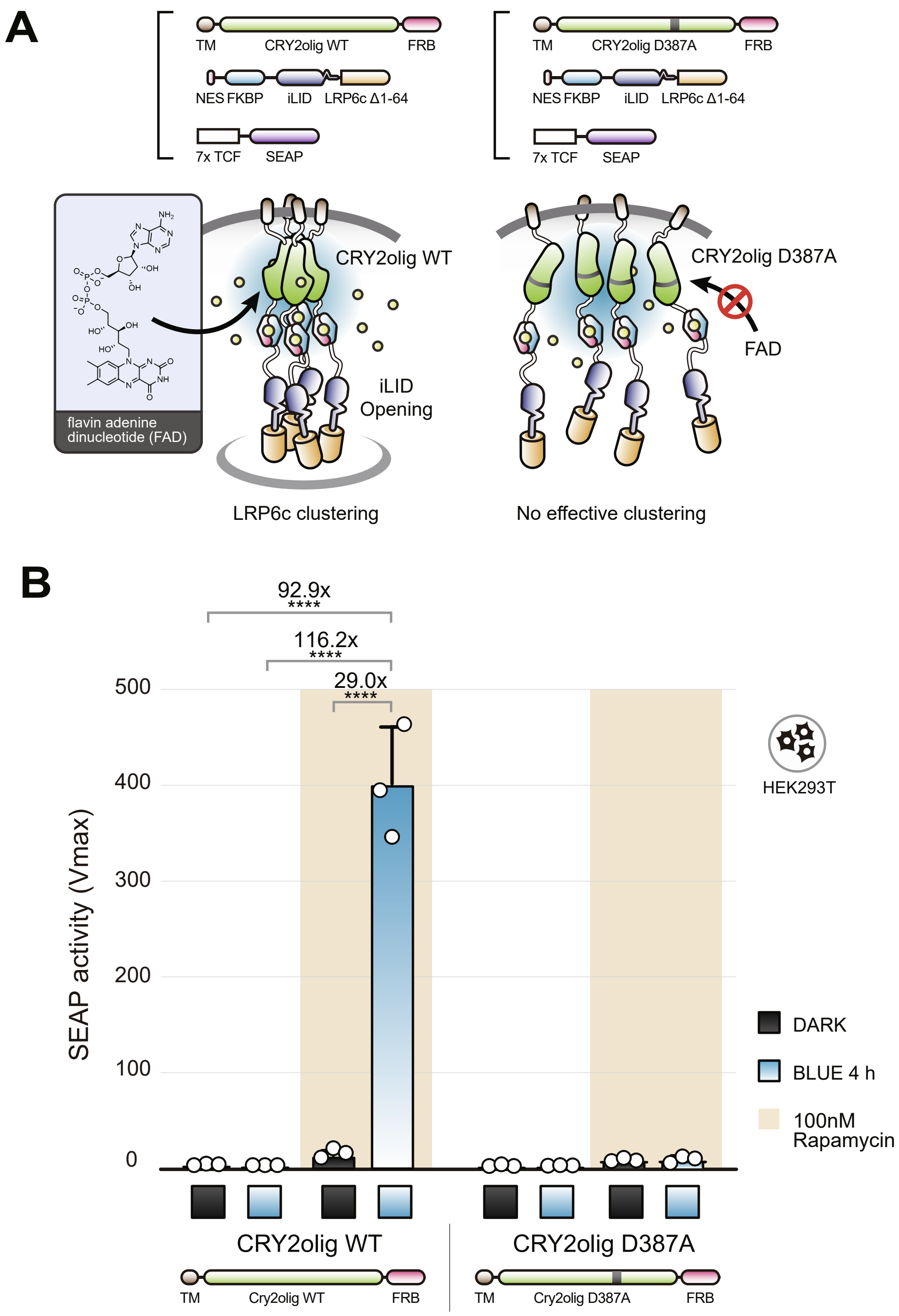

A
TM
CRY2olig WT
FRB
NES FKBP
iLID
LRP6c Δ1-64
7x TCF
SEAP
TM
CRY2olig D387A
FRB
NES FKBP
iLID
LRP6c Δ1-64
7x TCF
SEAP
flavin adenine dinucleotide (FAD)
CRY2olig WT
iLID Opening
LRP6c clustering
CRY2olig D387A
FAD
No effective clustering
B
92.9x
****
116.2x
****
29.0x
****
SEAP activity (Vmax)
500
400
300
200
100
0
HEK293T
DARK
BLUE 4 h
100nM Rapamycin
CRY2olig WT
TM
Cry2olig WT
FRB
CRY2olig D387A
TM
Cry2olig D387A
FRB

Figure S1. The indispensability of CRY2olig clustering in the activation of optochemoWnt, related to Figure 2

(A) Plasmid configurations of CRY2olig WT (left panel) and a functionally-dead mutant of CRY2olig, D387A (right panel) to examine the indispensability of CRY2olig clustering in optochemoWnt. FAD is a cofactor that ensures the optogenetic activity of CRY2olig. The D387A mutant, which lacks FAD interaction, no longer induces blue light-dependent clustering.

(B) Summary graph representing optochemoWnt activities in the CRY2olig WT and D387A mutant. The CRY2olig WT exhibited an AND-gated pattern of Wnt activation, whereas the D387A mutant shows complete inhibition of optochemoWnt activity.

Data were collected from three biologically independent samples. Values of measurement are presented as means ±S.D. and white circles represent individual measurements. The significance of the differences between blue light/rapamycin vs. the others was tested using a two-way ANOVA followed by Tukey's multiple comparisons test. ****$p < 0.0001$.

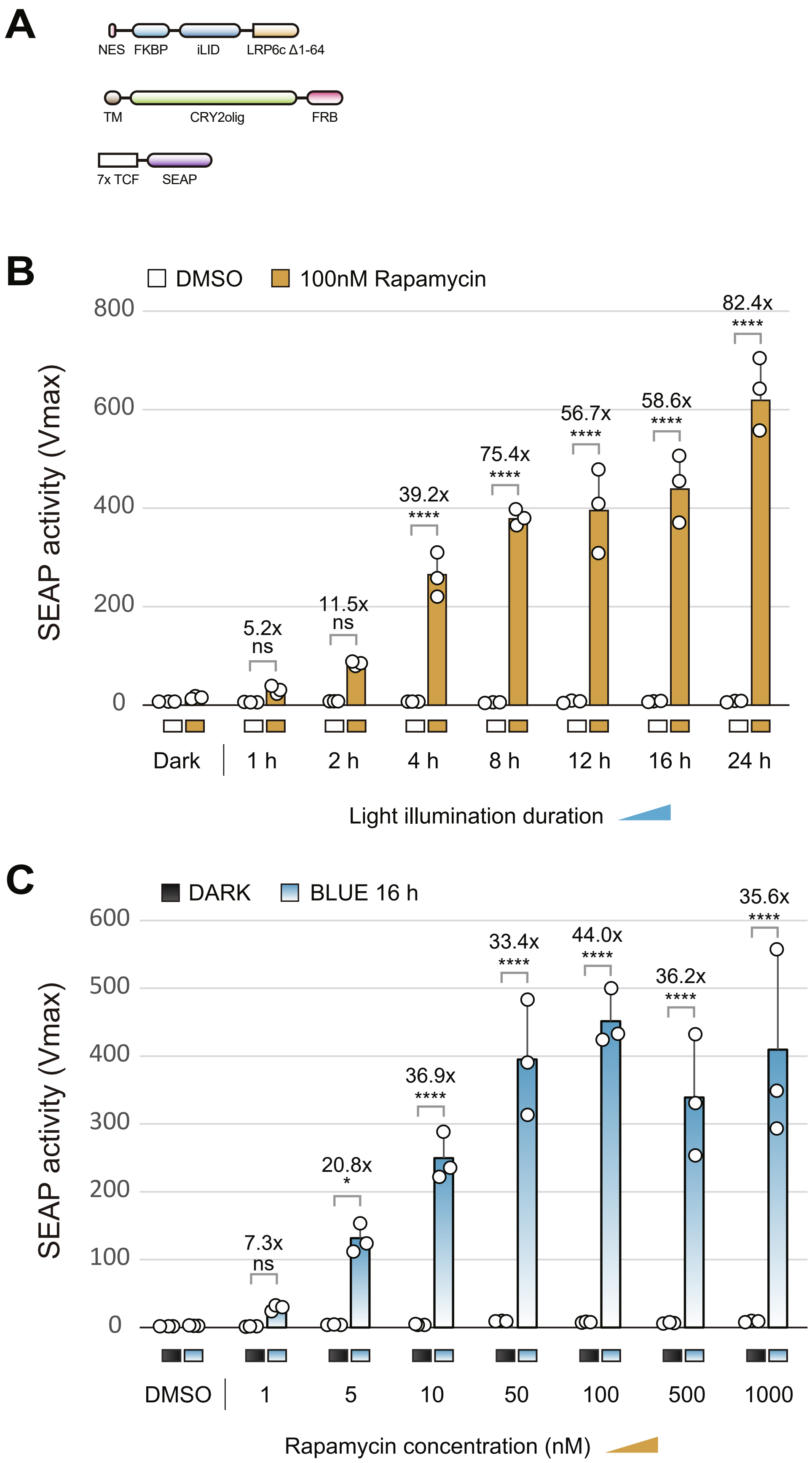
A
NES
FKBP
iLID
LRP6c Δ1-64
TM
CRY2olig
FRB
7x TCF
SEAP
B
DMSO
100nM Rapamycin
SEAP activity (Vmax)
800
600
400
200
0
5.2x
ns
11.5x
ns
39.2x
****
75.4x
****
56.7x
****
58.6x
****
82.4x
****
Dark
1 h
2 h
4 h
8 h
12 h
16 h
24 h
Light illumination duration
C
DARK
BLUE 16 h
SEAP activity (Vmax)
600
500
400
300
200
100
0
7.3x
ns
20.8x
*
36.9x
****
33.4x
****
44.0x
****
36.2x
****
35.6x
****
DMSO
1
5
10
50
100
500
1000
Rapamycin concentration (nM)

Figure S2. Characterization of optochemoWnt depending on optical duration and chemical concentration, related to Figure 3

(A) Configurations of optochemoWnt to characterize the optimal duration and concentration for optical and chemical stimulation.

(B) Characterization of induced gene expression versus light duration in optochemoWnt. The cells were grouped into 1 h, 2 h, 4 h, 8 h, 12 h, 16 h, and 24 h including dark conditions depending on the duration of blue light illumination. The concentration of rapamycin was fixed at 100 nM. The values are expressed as means ± S.D. and white circles are presented as individual measurements. Statistics included a two-way ANOVA followed by Tukey's multiple comparisons test. (ns = not significant, *$p < 0.05$).

(C) Characterization of induced gene expression versus rapamycin concentration in optochemoWnt. The dose-dependent chemical stimulation of optochemoWnt was analyzed at various concentrations (1 nM, 5 nM, 10 nM, 50 nM, 100 nM, 500 nM, and 1 μM). The duration of blue light was fixed at 16 h. SEAP expression of optochemoWnt peaked at 100 nM rapamycin. Data were collected from the three biologically independent samples and the white circles indicate individual measurements. The data are presented as means ± S.D. from three independent experiments. Statistics used included a two-way ANOVA followed by Tukey's multiple comparisons test (ns = not significant, ** = $p < 0.01$, **** = $p < 0.0001$).

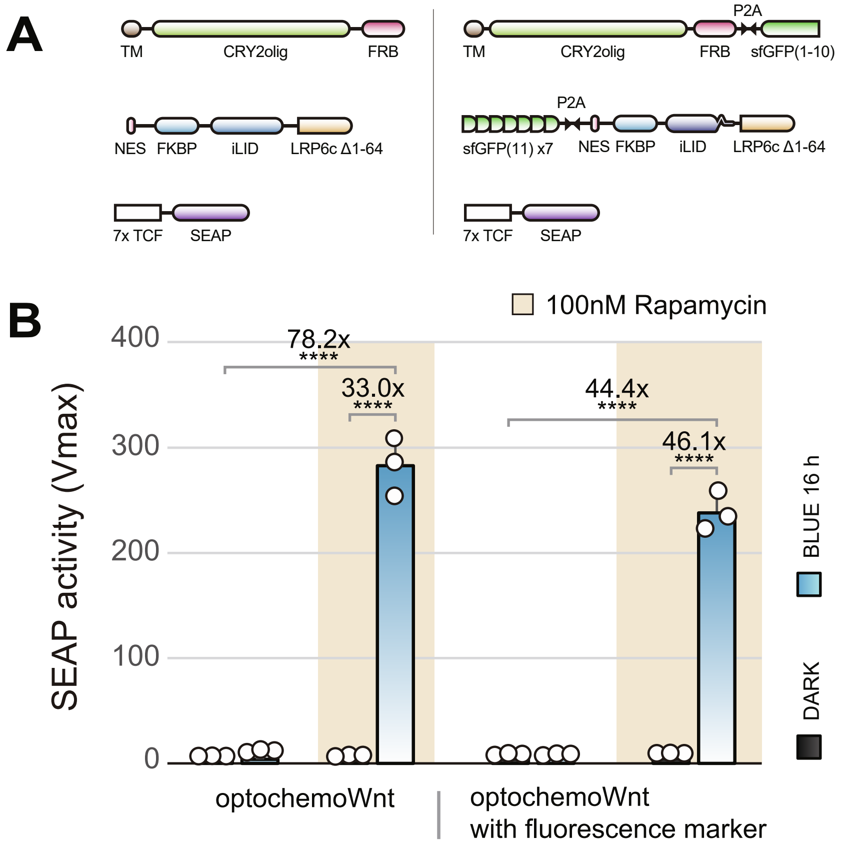

A
TM
CRY2olig
FRB
NES
FKBP
iLID
LRP6c Δ1-64
7x TCF
SEAP
P2A
sfGFP(1-10)
sfGFP(11) x7
B
100nM Rapamycin
SEAP activity (Vmax)
400
300
200
100
0
78.2x
****
33.0x
44.4x
46.1x
BLUE 16 h
DARK
optochemoWnt
optochemoWnt
with fluorescence marker

Figure S3. Performance comparison of optochemoWnt depending on the size of plasmid vectors , related to Figure 3

(A) Configurations of optochemoWnt and optochemoWnt with split spGFP.

(B) The gene expression level of optochemoWnt depending on the size of plasmid vectors. Data were collected from three biologically independent samples. The white circles represent individual measurements from three independent experiments. Two-way ANOVA followed by Tukey's multiple comparisons test. (****p<0.0001).
